# Supplementary material for: A Pathway to Precision Medicine for Aboriginal Australians: A Study Protocol
Source: Methods Protoc. 2021 Jun 21;4(2):42. doi: 10.3390/mps4020042 (PMC8293410; doi:10.3390/mps4020042)
Supplement: Supplementary file 1 [file mps-04-00042-s001.zip › mps-1250372-supplementary.pdf]

SUPPLEMENT 1- Initial Survey

Aboriginal Precision Medicine Initial Survey Questions

1. What is your age?

2. Are you Aboriginal?
   Yes
   No

3. Do you live in or around Glen Innes?
   Yes
   No

4. Are you
   Male
   Female
   Prefer not to answer

5. Have you watched the Personalised Medicine video?
   Yes
   No

6. Do you have any thoughts about the Project?
   Yes
   No
   Not sure

7. Do you think it is a good idea to collect samples in Glen Innes for the DNA Library (these will be anonymous)?
   Yes
   No

8. What did you think of the video?
   It helped me understand the project
   I found it confusing
   Not sure

SUPPLEMENT 2- Sample Letter of Support

AH&MRC ETHICS COMMITTEE
MODEL CONSENT FORM

ABORIGINAL COMMUNITY ORGANISATION

Name of Aboriginal Community Organisation:
(This must be an Aboriginal Community Controlled Health Service (ACCHS) unless otherwise approved by the Ethics Committee):

ARMAJUN ABORIGINAL HEALTH SERVICE

Project:

PERSONALISED MEDICINE

Principal Researcher: ASSOCIATE PROFESSOR KYLIE GWYNNE

Research Organisation: POCHE CENTRE FOR INDIGENOUS HEALTH
This must be completed by the Chairperson or CEO of the Aboriginal community organisation.

I, Debbie McCowen can confirm that ARMAJUN ABORIGINAL HEALTH SERVICE gives its consent to the above research project, subject to the following conditions:

1. We have the right to withdraw our consent and cease any further involvement in the research project at any time without any penalty and without giving any reasons.

2. The purpose of the research, as outlined in the attached brief, has been explained we have had the opportunity to ask questions about the project. We have received satisfactory answers to our questions and have been given adequate time to consider the appropriateness of the project.

3. We have been provided with the following information in writing:
   The names of all people and organisations that are responsible for the security of data and who will have access to the data.
   - Details of the proposed storage and destruction of data.

4. The researcher will need to obtain additional consent from us if there are any changes to the project from the information provided under paragraphs [2] and [3] above.

5. Any information that any member of our staff provides, or any personal details of our clients obtained in the course of this research, are confidential and any information that could identify individual participants will neither be used nor published.

6. Unless otherwise explicitly agreed, any information provided in the course of this research that identifies our organisation or the Aboriginal community which it serves will not be used nor published without our written permission.

7. The researcher will ensure there is continuing consultation with the community and our organisation during the course of the research. The research will not proceed until all required negotiation has occurred to our satisfaction.

8. The ethical provisions relating to the health of Aboriginal people, as set out in AH&MRC and NHMRC publications, will be complied with and the project will not proceed until the AH&MRC Ethics Committee has endorsed the project.

9. The researchers will obtain the written individual consent of all participants in the research.

10. We understand that if we have any complaints or questions concerning this research project, we can contact the principal researcher mentioned above; the Chairperson or CEO, or the Chairperson of the AH&MRC Ethics Committee as follows:

    The Chairperson
    AH&MRC Ethics Committee
    PO Box 1565
    Strawberry Hills NSW 2012
    Telephone: 9212 4777

Signed on AR IGINAL HEALTH SERVICE

Signature

As the Chief in the project, I acknowledge the conditions set out above

Name: ....

Signature..... *Date* 27/5/19

Witnessed by *Date* 2.

SUPPLEMENT 3- Consent form for individual participants

AH&MRC ETHICS COMMITTEE
MODEL CONSENT FORM

INDIVIDUAL PARTICIPANT

Project: ……………………………………………………………………………………

……………………………………………………………………………………………

Principal Researcher: ………………………………………………………………………

Research Organisation: ……………………………………………………………………

I, ……………………………… ……..………………………………………………………
have consented to participate in the above research project on the following basis:

1. I have received the Participant Information Statement and have had the opportunity to ask questions. I understand the purpose of the research and my involvement in it.

2. I have the right to withdraw my consent and cease any further involvement in the research project at any time without giving reasons and without any penalty. This will not affect any services that I receive.

3. Any information I provide during the course of this research will remain confidential. Where the results of the research are published, my involvement and my personal results will not be identified

4. I understand that interviews may be audio-taped or videotaped, but the tapes will be secured and then destroyed at the completion of the project.

5. I understand that if I have any complaints or questions concerning this research project I can contact the principal researcher, the Chairperson or CEO of the local Aboriginal Community Controlled Health Service; or the Chairperson of the AH&MRC Ethics Committee as follows:

   The Chairperson
   AH&MRC Ethics Committee
   P.O. Box 1565
   Strawberry Hills NSW 2012
   Telephone: 9212 4777

Name: …………………………………………………………………………………………

Signature …………………………………………… *Date* ………….………………..
Witnessed by ……………………………..……... *Date* …………………………....

Researcher’s signature : ……………………………………………
Date …..…………………

SUPPLEMENT 4- Plain English summary of the precision medicine project

ABORIGINAL PERSONALISED MEDICINE
Communication 02 – Protocol Summary

What's happened since we met last?

The partners have put together a grant application to the Australian Research Council to fund this project. In doing so, we have worked out some more details including how old people will need to be to participate in the study, the TAFE training courses, the way we will collect blood and how much the project will cost.

Everyone is very excited to get started.

In preparing the grant we have to write what is called "a protocol". A summary of the protocol is provided below. We would like your advice on the protocol. Some of it will be familiar because they are ideas we discussed last time we met.

How will we do this?

This research will be a partnership between the community and the research team and Armajun – we call this co-design. Together we will develop a culturally safe model of sample and data collection, governance, consent, data analysis and sharing results. Once we have agreed on the model, we will seek ethics approval from the Aboriginal Health and Medical Research Council Ethics Committee. We will need letters from Armajun and the Local Aboriginal Land Council to support the ethics application. Once data has been collected in the community, we will invite two other communities (one in rural and one urban) to participate.

How will recruit participants?

We will recruit 200 participants. We propose to host a three-day event at the Glenn Innes Aboriginal Land Council and Aboriginal Community Controlled Health Service. Over the three days information about genetics, the project, risks and benefits of the research will be presented by our research team.

A genetic counsellor (a counsellor who knows about DNA and how it impacts on people and families) will be available for each of the three days. Each participant will sign a form that says they are happy to be included in the research. An Aboriginal Pathology Collection Trainee will explain the form and answer any questions.

A key part of this project is creating opportunities for local people to become an Aboriginal Pathology Collection Trainees. This will mean undertaking a Certificate III in Pathology Collection and having special training in the protocol and ethics. The Poche Centre will provide scholarships for local Aboriginal people to undertake the Certificate III.

How will we collect the samples?

The samples will be collected by an Aboriginal Pathology Collection Trainee. The Trainees will be supervised by a senior scientist. Blood samples will be collected with a needle and saliva samples will be collected in saliva collection and storage units.

All samples will be prepared for sequencing and subsequently stored at the NSW Biobank. Information including location, sex, age and Aboriginal clan will accompany each deidentified sample. Access to the samples will be restricted to those approved by the Aboriginal Health and Medical Research Council Ethics Committee. The DNA stories from this community will be joined together as a single DNA book and will be added to the International DNA library.

This is an example of what the story of the DNA in the International DNA library will look like:

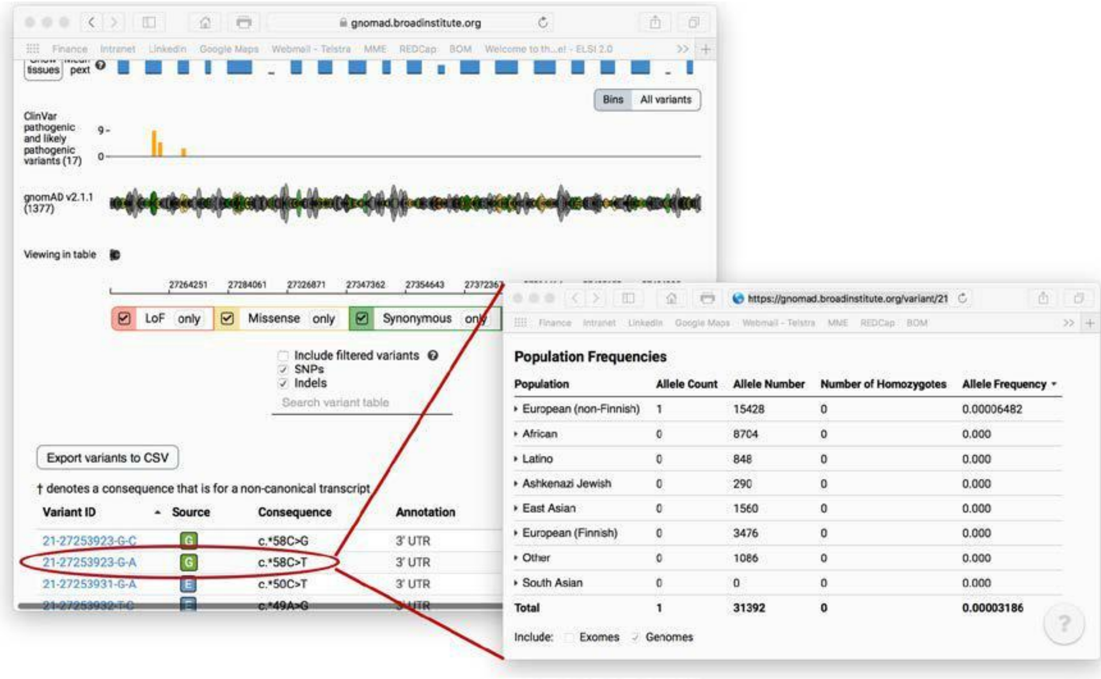

Who will analyse the samples?

Your DNA story will be sequenced from your sample, this will be undertaken by the Ramaciotti Centre for Genomics – who are very experienced in this work.

Research Governance

The research team will be guided by an Advisory Committee. The Advisory Committe e will meet at least three times a year. They will provide guidance to the research team on culturally safe research methods, community engagement and reporting of results of the project to participants. Community members will be invited to join the Advisory Committee and will have an active role.

What are the ethical considerations?

This research will be approved by the Ethics Committees of the Aboriginal Health and Medical Research Council (AHMRC) and the University of Sydney.

A short, animated video about the project has also been developed based on some existing work from the National Centre for Indigenous Genomics (NCIG) at ANU (https://youtu.be/S1dsAHONDBw). This was shown at the last community engagement forum.

Who are the Investigators?

The research team is a mix of Aboriginal and non-Aboriginal scholars with proven skills, experience and relationships to manage this project in NSW.

How does this project fit with other projects in Australia?

This project is significant as it focuses on NSW Aboriginal communities and will align strategically with similar projects underway with Indigenous communities of Western Australia, Queensland and the Northern Territory.

The goals of this project will align with other national efforts to enable genomic medicine for Aboriginal Australians including the National Centre for Indigenous Genomics at ANU. This national work does not currently include NSW Aboriginal communities.

How will the project benefit Indigenous Communities?

This project will mean there are more DNA stories to put into the larger library. This has the potential to improve diagnosis, tailor anti-cancer therapeutics, and enable targeted personalised medicine for rare genetic conditions.

How will the results be communicated?

We will share the results of the project through the Advisory Committee, community meetings and newsletters.

How will my data be managed?

Data resulting from the sequencing will be stored at the University of Sydney and/or the NCI in a secure environment in keeping the requirements of the University of Sydney and the Aboriginal Health and Medical Research Council Ethics Committees.

As part of the project a data access policy will be developed in conjunction with partners and Aboriginal community representatives on the Project Advisory Committee. It is likely to include three tiers of data access: open access, controlled access and restricted access. The translational and clinical implementation of this Reference Genome is outside the scope and funding of this grant.

Who is involved in this project?

This project has four teams collaborating: Kids Research Institute (KRI) at Westmead, National Centre for Indigenous Genomics, Australian Genomics and the Poche Centre for Indigenous Health, University of Sydney (Poche Centre).

Poche Centre for Indigenous Health
poche.admin@sydney.edu.au | 02 9114 0829

Armajun Aboriginal Health Service
info@armajun.org.au | (02) 6721 9777
